# Physiological Observations on the Transformations of the Organs of the Human Body

**Published:** 1806-10

**Authors:** C. L. Dumas

**Affiliations:** the Imperial Institute, Professor in the School of Medicine at Montpellier, &c. &c.


*Physiological Observations on the Transformations of the Organs of the Human Body. By* C. L. DUMAS, *of the Imperial Institute, Professor in the School of Medicine at Montpellier, &c. &c.*

AMONG the changes to which the organs of the human body are liable, many examples of which may be found in anatomical publications, none appears more remarkable than those which, by a kind of transmutation in their form and composition, render them similar to other parts, with which they originally had no analogy.

The cartilages ossify, the muscles change into a fatty substance, the tendons are converted into membranes, the lungs assume the form and consistence of the liver, the mucous membranes, and the parenchymatous substance of the viscera acquire the softness of the pulpy matter of the brain, the cellular membrane becomes condensed, shrivelled, and dry, like the epidermis, the brain changes into a solid, osseous, or indurated mass; such are the extraordinary phenomena which the transformation of the organs presents to our view. The works of Bonnet, Morgagni, and Lieutaud, as well as the transactions of many learned Societies, contain a multitude of facts of this kind, which only require arrangement and classification.

In such singular transformations, the organ, which changes its character to assume that of another, acquires the structure, the composition, and properties of the latter. The order and succession of the causes, which concur to produce this phenomenon, are neither sufficiently known, nor understood. Sufficient data are still wanting to explain, or even to class, all the facts relative to this species of conversion. It seems then important to multiply observations, and to mark the appearances, which dissections may furnish respecting those parts of the human body, which are most susceptible of such changes.

All the transformations of the organs may be produced by changes either in their physical and sensible qualities, in their constituent principles and chemical composition, in their form and structure, or in their functions and vital properties. These form four principal classes, under which may be arranged all organic transformations:

1st. Those which relate to their physical constitution.

2d. Those which relate to their chemical composition.

3d. Those which relate to their organic structure.

4th. Those which relate to their properties and vital functions.

An

An organ is susceptible of assuming the physical and external qualities of another organ, so that it shall resemble it in figure, size, colour, &c. This species of transformation may occur in the thoracic and abdominal viscera. In this manner, we have seen the spleen assume the colour and magnitude of the liver, the pancreas become equal in size to the larger viscera, the great arterial vessels expanding into muscular sacs, so as to put on the appearance of the heart. These transformations, which relate to the physical qualities of the organs, are common to the different parts of the digestive system. Morgagni mentions his having seen in several bodies the duodenum distended, and enlarged, to such a degree, that it appeared like a second stomach. I myself once witnessed the inferior portion of the œsophagus so dilated, that it resembled the first stomach of a ruminant animal.

2. The constituent principles which enter into the composition of an organ, in its natural state, may be transferred to another, and occasion such a change in its assimilating powers, as to convert it into the nature of the former organ. These transformations will produce different results, according to the nature of the substances thus transferred. Such changes, however, in general, proceed from four principle causes:

1st. From a deviation of the albuminous substance.

2d. From a deviation of the fibrous substance.

3d. From a deviation of gelatine.

4th. From a deviation of calcareous earthy salts.

Some organs derive their nourishment from the albuminous principle, and appear to be essentially composed of it; such as the brain and the greatest part of the abdominal viscera, in which albumen obviously predominates. But albuminous matter enters besides into the composition of many other organs, such as the muscles, cartilages, and ligaments, in which it is combined, though in an inferior degree, with other substances. Now it may happen, either that the albumen, which is necessary to the nutrition of these organs, may be taken in, and accumulated in too great a quantity, or that the other principles entering into combination with it, may acquire the albuminous character; in either case, the quantity of albuminous matter may become predominant, and approximate the organs, as far as regards their composition, to the nature of those parts which contain albumen in the greatest abundance.

On the other hand, it is known, that all the parts of the animal body become changed after death, into a fatty matter ter of an albuminous nature, analogous to spermaceti, known to chemists under the name of *adipocere*. This change may occur during life, and produce in the organs that species of conversion which should only occur after the extinction of life. Such are then the causes of the not unfrequent change of muscles and viscera, into a matter similar to adipocere, which is the last stage in the decomposition of dead animal bodies.

Besides the instances of such conversions with which the works of anatomists abound, I may here mention a case that I myself had occasion to witness, in which the muscles were completely changed into a substance perfectly resembling fat.—This patient had long been afflicted with a catarrhal fever, which, at last, terminated in death. On opening the body, we discovered that the muscles of the anterior part of the chest, as well as those of the posterior surface of the shoulder and arm, were reduced to a fatty mass contained in a sac of condensed cellular membrane, and exactly of the form and figure of the muscles, whose place it occupied. Some other muscles, as those of the lower belly, and the triceps cruralis, had not yet undergone a complete change, but their fibres, both in consistence and colour, indicated that this alteration would have speedily taken place. The glutaeus maximus, and a part of the adductor femoris, though partially changed, still exhibited a mixture of muscular fibres in the interstices of the fatty matter. I might adduce many other instances of this kind, which have fallen under my own observation, but this would be wholly superfluous, since so many analogous cases have been already noticed by anatomists.

May we not refer to this class of transformations, the spontaneous production of those organic masses that are formed in encysted tumors? Ought we not to attribute to the same cause the progressive softening of some fibrous organs, which are by degrees deprived of their solidity, and degenerate into a membranous or cellular substance? Thus we read in some anatomical publications, that the tendinous extremities of the muscles sometimes become so attenuated, as to resemble membranes; either from having lost the principle of solidity, or from being impregnated with the albuminous juices peculiar to serous membranes.

The fibrous matter of the blood, which enters immediately into the composition of the muscular organs, may be deposited on other parts, and impart to them, through a defect of assimilation, the fibrous and muscular character.

ter. It is thus that polypous concretions are formed, and that membranous and vascular organs are converted, by the effect of these multiplied concretions, into a hard, compact, fibrous substance, analogous to that of the muscles. Transformations of this kind, chiefly occur towards the conclusion of inflammatory affections, as if one of the sequelæ of inflammation was to render the fibrine predominant in parts attacked by it. If this degeneracy occur in a cellular organ, the fibrine must combine with the cellular substance, and by its combination produce an organization similar to the parenchyma of certain viscera.

In this way may be explained the remarkable conversion of the lungs into a granular, reddish, dense, grumous substance, analogous to that of the liver. Morgagni expresses this transformation of the pulmonary organs by saying, that they are changed into a liver-like substance; pulmonum substantia quasi in hepaticam mutata; pulmonem substantiæ factum hepaticæ similem; pulmo hepaticæ instar substantiæ. This conversion frequently supervenes, as I myself have witnessed, after inflammatory affections of the pulmonary organs. In my Principles of Physiology, the history of a dissection is given, in which the left lobe of the lungs did not retain the smallest trace of its original organization. Its size was enormous, and in colour, density, and figure, it resembled the liver; its substance was granular, thick, similar to coagulated blood, like the the substance of that organ; a yellow, thick, saponaceous fluid resembling bile in colour and consistence, constantly oozed from every point of the diseased organ.

As the solid parts of animal bodies admit gelatine into their composition only in combination with other principles, which determine their natural cohesion and solidity, it is evident, that the casual predominance of the gelatinous matter, though it may operate to alter the texture of the organs in which it is deposited in an undue quantity, cannot, however, impart to them characters analogous to the structure of other solids, which exist naturally formed in the animal body. Thus we see the most solid organs, such as the bones and cartilages, become occasionally so soft, as to indicate a superabundance of gelatine in their composition. It is besides not improbable, that this matter may sometimes be deposited in other parts, so as spontaneously to form mucous bodies, in which gelatine abounds.

( To be continued. )

